# Supplementary material for: Deletion of wfs1 Impairs Oligodendrocyte Precursor Cells Dorsal Distribution and Myelination Through the wfs1-hmgcs1 Axis in Zebrafish
Source: Biology (Basel). 2026 Aug 16;15(16):1402. doi: 10.3390/biology15161402 (PMC13510151; doi:10.3390/biology15161402)
Supplement: Supplementary file 1 [file biology-15-01402-s001.zip › File S1-Transcriptome sequencing data and differentially expressed genes (DEGs) in wfs1-- zebrafish at 5 dpf/2.KEGG/wfs1KOvsWT/GSEA/WT/NECROPTOSIS/NECROPTOSIS(DRE04217).html]

Details for gene set NECROPTOSIS(DRE04217)[GSEA]

|  || Dataset | deg.sample |
| Phenotype | sample.cls |
| Upregulated in class | WT |
| GeneSet | NECROPTOSIS(DRE04217) |
| Enrichment Score (ES) | -0.29231682 |
| Normalized Enrichment Score (NES) | -1.4299718 |
| Nominal p-value | 0.0 |
| FDR q-value | 0.53413063 |
| FWER p-Value | 0.853 |
Table: GSEA Results Summary

  

Fig 1: Enrichment plot: NECROPTOSIS(DRE04217)      
 Profile of the Running ES Score & Positions of GeneSet Members on the Rank Ordered List

  

| PROBE | DESCRIPTION (from dataset) | GENE SYMBOL | GENE\_TITLE | RANK IN GENE LIST | RANK METRIC SCORE | RUNNING ES | CORE ENRICHMENT || 1 | 337413\_1 | stat6 |  |  | 18 | 4.955 | 0.0334 | No |
| 2 | 566185 | caspbl |  |  | 88 | 4.759 | 0.0639 | No |
| 3 | 393186\_1 | zgc:56235 |  |  | 453 | 3.118 | 0.0737 | No |
| 4 | 110438871 | LOC110438871 |  |  | 575 | 2.799 | 0.0891 | No |
| 5 | 561021 | si:dkey-103e21.5 |  |  | 599 | 2.768 | 0.1074 | No |
| 6 | 336426 | chmp1b |  |  | 758 | 2.459 | 0.1192 | No |
| 7 | 393444 | pygmb |  |  | 1013 | 2.082 | 0.1255 | No |
| 8 | 100148591 | LOC100148591 |  |  | 1308 | 1.801 | 0.1286 | No |
| 9 | 768248 | fas |  |  | 1486 | 1.661 | 0.1344 | No |
| 10 | 565200\_2 | stat2 |  |  | 2159 | 1.302 | 0.1221 | No |
| 11 | 445091\_1 | zgc:92480 |  |  | 2191 | 1.289 | 0.1299 | No |
| 12 | 100149436 | si:ch211-189a15.5 |  |  | 2192 | 1.289 | 0.1388 | No |
| 13 | 100147988 | chmp6a |  |  | 2217 | 1.281 | 0.1468 | No |
| 14 | 373107 | birc2 |  |  | 2293 | 1.250 | 0.1530 | No |
| 15 | 108182827 | LOC108182827 |  |  | 2311 | 1.246 | 0.1610 | No |
| 16 | 560527 | si:ch211-113a14.19 |  |  | 2517 | 1.168 | 0.1625 | No |
| 17 | 798864\_1 | pla2g4f.1 |  |  | 2559 | 1.155 | 0.1692 | No |
| 18 | 678613 | h2afy |  |  | 3293 | 0.974 | 0.1527 | No |
| 19 | 564295\_1 | traf2a |  |  | 3833 | 0.862 | 0.1416 | No |
| 20 | 569532\_1 | chmp2ba |  |  | 3911 | 0.846 | 0.1450 | No |
| 21 | 795671\_1 | tlr4al |  |  | 4308 | 0.775 | 0.1378 | No |
| 22 | 553552 | zgc:109934 |  |  | 4319 | 0.773 | 0.1428 | No |
| 23 | 566370\_1 | slc25a6 |  |  | 4580 | 0.736 | 0.1396 | No |
| 24 | 100001092 | eif2ak2 |  |  | 4849 | 0.699 | 0.1359 | No |
| 25 | 565825 | si:dkeyp-50d11.2 |  |  | 4870 | 0.698 | 0.1401 | No |
| 26 | 559087 | pla2g4ab |  |  | 4877 | 0.697 | 0.1447 | No |
| 27 | 100004321 | LOC100004321 |  |  | 5124 | 0.665 | 0.1415 | No |
| 28 | 565200 | stat2 |  |  | 5154 | 0.661 | 0.1451 | No |
| 29 | 100000783 | si:ch73-368j24.14 |  |  | 5398 | 0.640 | 0.1418 | No |
| 30 | 565386\_1 | pla2g4f.2 |  |  | 5440 | 0.638 | 0.1449 | No |
| 31 | 100148581 | LOC100148581 |  |  | 5502 | 0.634 | 0.1473 | No |
| 32 | 564295 | traf2a |  |  | 5504 | 0.634 | 0.1516 | No |
| 33 | 334582 | vdac1 |  |  | 5612 | 0.625 | 0.1525 | No |
| 34 | 327067 | slc25a4 |  |  | 5868 | 0.600 | 0.1486 | No |
| 35 | 735295 | faslg |  |  | 6149 | 0.572 | 0.1437 | No |
| 36 | 798222 | camk2b |  |  | 6413 | 0.544 | 0.1391 | No |
| 37 | 100332229 | LOC100332229 |  |  | 6524 | 0.535 | 0.1393 | No |
| 38 | 317737\_1 | glud1a |  |  | 6910 | 0.499 | 0.1306 | No |
| 39 | 65236 | mapk8b |  |  | 6998 | 0.492 | 0.1312 | No |
| 40 | 58130 | tradd |  |  | 7033 | 0.489 | 0.1335 | No |
| 41 | 57923 | pycard |  |  | 7045 | 0.488 | 0.1365 | No |
| 42 | 30554 | pla2g4aa |  |  | 7169 | 0.476 | 0.1358 | No |
| 43 | 100001004 | si:ch73-368j24.15 |  |  | 7480 | 0.455 | 0.1292 | No |
| 44 | 795671 | tlr4al |  |  | 7485 | 0.455 | 0.1322 | No |
| 45 | 793359\_1 | camk2g2 |  |  | 7602 | 0.447 | 0.1316 | No |
| 46 | 798864 | pla2g4f.1 |  |  | 7791 | 0.438 | 0.1286 | No |
| 47 | 447823 | fth1b |  |  | 7921 | 0.429 | 0.1275 | No |
| 48 | 360134 | ifnphi1 |  |  | 9074 | 0.362 | 0.0936 | No |
| 49 | 566871 | si:dkey-32e23.4 |  |  | 9099 | 0.360 | 0.0953 | No |
| 50 | 373108 | xiap |  |  | 9360 | 0.343 | 0.0895 | No |
| 51 | 336473\_1 | glulb |  |  | 9542 | 0.330 | 0.0860 | No |
| 52 | 793359 | camk2g2 |  |  | 9631 | 0.325 | 0.0855 | No |
| 53 | 793673 | sharpin |  |  | 9691 | 0.322 | 0.0858 | No |
| 54 | 565155 | hsp90aa1.2 |  |  | 9737 | 0.319 | 0.0866 | No |
| 55 | 562720 | mapk8a |  |  | 9888 | 0.310 | 0.0840 | No |
| 56 | 557526 | traf2b |  |  | 10340 | 0.282 | 0.0717 | No |
| 57 | 403051\_1 | pygb |  |  | 11510 | 0.216 | 0.0363 | No |
| 58 | 405785 | tnfa |  |  | 11720 | 0.205 | 0.0311 | No |
| 59 | 393386 | cybb |  |  | 11782 | 0.202 | 0.0305 | No |
| 60 | 100334178 | LOC100334178 |  |  | 11935 | 0.194 | 0.0271 | No |
| 61 | 559425 | bida |  |  | 12011 | 0.190 | 0.0260 | No |
| 62 | 100003969 | zgc:165551 |  |  | 12205 | 0.179 | 0.0211 | No |
| 63 | 557302 | casp8l2 |  |  | 12635 | 0.157 | 0.0087 | No |
| 64 | 558613 | chmp5a |  |  | 12765 | 0.151 | 0.0056 | No |
| 65 | 403013 | irf9 |  |  | 12801 | 0.150 | 0.0055 | No |
| 66 | 560788 | parp1 |  |  | 13023 | 0.139 | -0.0005 | No |
| 67 | 100049706 | casp8l1 |  |  | 13088 | 0.136 | -0.0016 | No |
| 68 | 562064 | camk2b1 |  |  | 13117 | 0.134 | -0.0015 | No |
| 69 | 568830 | spata2 |  |  | 13833 | 0.100 | -0.0234 | No |
| 70 | 30280 | jak1 |  |  | 13843 | 0.100 | -0.0230 | No |
| 71 | 403077 | h2afvb |  |  | 13875 | 0.098 | -0.0233 | No |
| 72 | 569698 | mapk10 |  |  | 13907 | 0.096 | -0.0236 | No |
| 73 | 445397 | chmp6b |  |  | 14023 | 0.091 | -0.0266 | No |
| 74 | 403132\_1 | tlr4bb |  |  | 14024 | 0.091 | -0.0260 | No |
| 75 | 405770 | il1b |  |  | 14290 | 0.079 | -0.0339 | No |
| 76 | 406243 | faf1 |  |  | 14415 | 0.073 | -0.0373 | No |
| 77 | 569903 | shrprbck1r |  |  | 14645 | 0.063 | -0.0441 | No |
| 78 | 100038774 | si:dkey-251i10.1 |  |  | 14670 | 0.062 | -0.0444 | No |
| 79 | 58081 | baxa |  |  | 14773 | 0.057 | -0.0472 | No |
| 80 | 58022 | casp8 |  |  | 15145 | 0.039 | -0.0587 | No |
| 81 | 403147 | ticam1 |  |  | 15198 | 0.037 | -0.0601 | No |
| 82 | 280653 | trpm7 |  |  | 15717 | 0.010 | -0.0763 | No |
| 83 | 445474 | stat5b |  |  | 15905 | 0.002 | -0.0822 | No |
| 84 | 394048 | h2afx |  |  | 15917 | 0.002 | -0.0826 | No |
| 85 | 563053 | capn2b |  |  | 15938 | 0.001 | -0.0832 | No |
| 86 | 560309 | si:ch211-113a14.16 |  |  | 15993 | -0.001 | -0.0849 | No |
| 87 | 30767 | stat3 |  |  | 16064 | -0.004 | -0.0871 | No |
| 88 | 100006331 | si:dkey-23a13.14 |  |  | 16118 | -0.006 | -0.0887 | No |
| 89 | 100006523 | zgc:198419 |  |  | 16166 | -0.007 | -0.0901 | No |
| 90 | 565825\_1 | si:dkeyp-50d11.2 |  |  | 16172 | -0.008 | -0.0902 | No |
| 91 | 565386 | pla2g4f.2 |  |  | 16232 | -0.009 | -0.0920 | No |
| 92 | 100005446 | LOC100005446 |  |  | 16477 | -0.017 | -0.0996 | No |
| 93 | 554094 | zgc:110434 |  |  | 16513 | -0.017 | -0.1006 | No |
| 94 | 103911344 | si:ch1073-159d7.9 |  |  | 16564 | -0.019 | -0.1021 | No |
| 95 | 373125\_1 | aifm1 |  |  | 16604 | -0.020 | -0.1032 | No |
| 96 | 570772 | bcl2a |  |  | 16638 | -0.021 | -0.1041 | No |
| 97 | 393880 | vps4b |  |  | 17091 | -0.036 | -0.1181 | No |
| 98 | 30591 | hsp90aa1.1 |  |  | 17250 | -0.043 | -0.1228 | No |
| 99 | 561370 | jak3 |  |  | 17265 | -0.044 | -0.1229 | No |
| 100 | 393351 | chmp5b |  |  | 17275 | -0.044 | -0.1229 | No |
| 101 | 100003387 | spata2l |  |  | 17658 | -0.061 | -0.1345 | No |
| 102 | 100149273 | mapk9 |  |  | 17865 | -0.070 | -0.1406 | No |
| 103 | 767670 | vps4a |  |  | 17948 | -0.073 | -0.1427 | No |
| 104 | 406529 | vdac3 |  |  | 17989 | -0.075 | -0.1434 | No |
| 105 | 30280\_1 | jak1 |  |  | 18082 | -0.080 | -0.1458 | No |
| 106 | 30298 | jak2b |  |  | 18488 | -0.098 | -0.1579 | No |
| 107 | 767645 | zgc:153993 |  |  | 18516 | -0.099 | -0.1580 | No |
| 108 | 556700 | smpd1 |  |  | 18519 | -0.100 | -0.1574 | No |
| 109 | 564497 | tnfaip3 |  |  | 18538 | -0.100 | -0.1573 | No |
| 110 | 100329124 | ifngr1 |  |  | 18577 | -0.102 | -0.1578 | No |
| 111 | 415155 | ppid |  |  | 18744 | -0.111 | -0.1623 | No |
| 112 | 393535 | chmp1a |  |  | 18849 | -0.116 | -0.1648 | No |
| 113 | 406766 | chmp4bb |  |  | 19021 | -0.122 | -0.1693 | No |
| 114 | 561187 | si:ch211-113a14.26 |  |  | 19116 | -0.127 | -0.1714 | No |
| 115 | 100000775 | glula |  |  | 19256 | -0.133 | -0.1749 | No |
| 116 | 795095 | hmgb1b |  |  | 19300 | -0.135 | -0.1753 | No |
| 117 | 393164 | chmp4ba |  |  | 19448 | -0.142 | -0.1790 | No |
| 118 | 556823 | cylda |  |  | 19626 | -0.149 | -0.1836 | No |
| 119 | 337730\_1 | capn8 |  |  | 19702 | -0.153 | -0.1849 | No |
| 120 | 492357 | pgam5 |  |  | 19705 | -0.153 | -0.1839 | No |
| 121 | 30573 | hsp90ab1 |  |  | 19796 | -0.157 | -0.1857 | No |
| 122 | 58130\_1 | tradd |  |  | 19801 | -0.157 | -0.1847 | No |
| 123 | 541374 | capn2a |  |  | 19921 | -0.163 | -0.1874 | No |
| 124 | 791855 | camk2g1 |  |  | 20264 | -0.177 | -0.1969 | No |
| 125 | 406471 | tnfrsf1a |  |  | 20265 | -0.178 | -0.1957 | No |
| 126 | 100334934 | LOC100334934 |  |  | 20272 | -0.178 | -0.1947 | No |
| 127 | 567642 | si:dkey-181m9.8 |  |  | 20372 | -0.182 | -0.1966 | No |
| 128 | 405790 | ifng1-2 |  |  | 20411 | -0.183 | -0.1965 | No |
| 129 | 373093 | hdr |  |  | 20727 | -0.200 | -0.2051 | No |
| 130 | 406621 | chmp3 |  |  | 20867 | -0.208 | -0.2080 | No |
| 131 | 373092 | glud1b |  |  | 20882 | -0.209 | -0.2071 | No |
| 132 | 563887 | traf5 |  |  | 20919 | -0.211 | -0.2067 | No |
| 133 | 492789 | zgc:101699 |  |  | 20960 | -0.213 | -0.2065 | No |
| 134 | 553655 | pygma |  |  | 21309 | -0.231 | -0.2160 | No |
| 135 | 558711 | h2afy2 |  |  | 21397 | -0.236 | -0.2171 | No |
| 136 | 393896 | dnm1l |  |  | 21645 | -0.248 | -0.2232 | No |
| 137 | 406286 | zgc:56095 |  |  | 21686 | -0.251 | -0.2227 | No |
| 138 | 436866 | tnfsf10 |  |  | 21725 | -0.253 | -0.2222 | No |
| 139 | 100004768 | si:dkey-261m9.7 |  |  | 21752 | -0.254 | -0.2213 | No |
| 140 | 192321 | slc25a5 |  |  | 21812 | -0.257 | -0.2214 | No |
| 141 | 566165 | glulc |  |  | 21829 | -0.258 | -0.2201 | No |
| 142 | 252913 | h2afva |  |  | 21852 | -0.259 | -0.2190 | No |
| 143 | 405840 | chmp2bb |  |  | 22045 | -0.270 | -0.2232 | No |
| 144 | 373114 | cflara |  |  | 22061 | -0.270 | -0.2219 | No |
| 145 | 100125271 | zgc:173652 |  |  | 22069 | -0.271 | -0.2202 | No |
| 146 | 559220 | si:dkey-261m9.15 |  |  | 22153 | -0.276 | -0.2209 | No |
| 147 | 30307 | jak2a |  |  | 22173 | -0.277 | -0.2196 | No |
| 148 | 322126 | vdac2 |  |  | 22259 | -0.282 | -0.2204 | No |
| 149 | 550436 | camk2a |  |  | 22386 | -0.289 | -0.2224 | No |
| 150 | 559425\_1 | bida |  |  | 22391 | -0.289 | -0.2205 | No |
| 151 | 100334621 | si:ch73-36p18.5 |  |  | 22544 | -0.298 | -0.2233 | No |
| 152 | 335519 | ppiab |  |  | 22648 | -0.305 | -0.2245 | No |
| 153 | 406452 | sqstm1 |  |  | 22703 | -0.308 | -0.2241 | No |
| 154 | 561108 | cyldl |  |  | 23056 | -0.331 | -0.2329 | No |
| 155 | 567460 | ripk1l |  |  | 23090 | -0.333 | -0.2317 | No |
| 156 | 58108 | fth1a |  |  | 23202 | -0.341 | -0.2328 | No |
| 157 | 445091 | zgc:92480 |  |  | 23577 | -0.365 | -0.2421 | No |
| 158 | 565106 | capn1b |  |  | 23645 | -0.370 | -0.2417 | No |
| 159 | 369197\_1 | stat5a |  |  | 23647 | -0.370 | -0.2392 | No |
| 160 | 563546 | usp21 |  |  | 23670 | -0.371 | -0.2373 | No |
| 161 | 30768 | stat1a |  |  | 23920 | -0.387 | -0.2425 | No |
| 162 | 100004555 | ripk3 |  |  | 23949 | -0.389 | -0.2408 | No |
| 163 | 100004011 | zgc:195633 |  |  | 24330 | -0.415 | -0.2499 | No |
| 164 | 393754 | chmp7 |  |  | 24474 | -0.418 | -0.2516 | No |
| 165 | 393417 | capn1a |  |  | 24498 | -0.419 | -0.2494 | No |
| 166 | 100000574 | si:ch73-368j24.13 |  |  | 24842 | -0.440 | -0.2572 | No |
| 167 | 317737 | glud1a |  |  | 25006 | -0.446 | -0.2593 | No |
| 168 | 798901 | bc2 |  |  | 25024 | -0.448 | -0.2568 | No |
| 169 | 570115 | LOC570115 |  |  | 25132 | -0.456 | -0.2570 | No |
| 170 | 101882533 | si:ch211-113a14.7 |  |  | 25156 | -0.457 | -0.2546 | No |
| 171 | 100538134 | si:dkey-261m9.18 |  |  | 25159 | -0.457 | -0.2516 | No |
| 172 | 553353 | si:ch211-202f3.3 |  |  | 25417 | -0.477 | -0.2564 | No |
| 173 | 57933 | caspa |  |  | 25658 | -0.499 | -0.2606 | No |
| 174 | 100332885 | si:dkey-108k21.26 |  |  | 25816 | -0.512 | -0.2620 | No |
| 175 | 436815 | camk2d2 |  |  | 25880 | -0.518 | -0.2604 | No |
| 176 | 562317 | fadd |  |  | 26068 | -0.535 | -0.2627 | No |
| 177 | 768248\_1 | fas |  |  | 26075 | -0.536 | -0.2592 | No |
| 178 | 406768 | zgc:55262 |  |  | 26113 | -0.538 | -0.2567 | No |
| 179 | 103909881 | si:dkey-108k21.15 |  |  | 26241 | -0.551 | -0.2569 | No |
| 180 | 449794 | zgc:101846 |  |  | 26714 | -0.600 | -0.2677 | No |
| 181 | 445208 | camk2d1 |  |  | 26863 | -0.615 | -0.2681 | No |
| 182 | 110439839 | LOC110439839 |  |  | 27117 | -0.641 | -0.2717 | No |
| 183 | 100536675 | tyk2 |  |  | 27404 | -0.658 | -0.2763 | No |
| 184 | 777653 | crfb2 |  |  | 27470 | -0.669 | -0.2737 | No |
| 185 | 100170833 | zgc:194125 |  |  | 27775 | -0.712 | -0.2784 | No |
| 186 | 493916 | pygl |  |  | 28092 | -0.756 | -0.2832 | No |
| 187 | 403126\_1 | tlr3 |  |  | 28163 | -0.766 | -0.2802 | No |
| 188 | 403131\_1 | tlr4ba |  |  | 28357 | -0.796 | -0.2808 | No |
| 189 | 368519 | stat4 |  |  | 28722 | -0.863 | -0.2864 | Yes |
| 190 | 403051 | pygb |  |  | 28772 | -0.872 | -0.2820 | Yes |
| 191 | 393186 | zgc:56235 |  |  | 28953 | -0.909 | -0.2814 | Yes |
| 192 | 431715 | rbck1 |  |  | 29001 | -0.920 | -0.2766 | Yes |
| 193 | 566370 | slc25a6 |  |  | 29069 | -0.936 | -0.2723 | Yes |
| 194 | 100536331 | si:ch211-113a14.11 |  |  | 29096 | -0.941 | -0.2667 | Yes |
| 195 | 100007048 | bcl2b |  |  | 29242 | -0.987 | -0.2645 | Yes |
| 196 | 436651 | zgc:92066 |  |  | 29314 | -1.005 | -0.2598 | Yes |
| 197 | 561021\_1 | si:dkey-103e21.5 |  |  | 29577 | -1.086 | -0.2607 | Yes |
| 198 | 369197 | stat5a |  |  | 29591 | -1.090 | -0.2536 | Yes |
| 199 | 373125 | aifm1 |  |  | 30075 | -1.274 | -0.2601 | Yes |
| 200 | 337730 | capn8 |  |  | 30080 | -1.277 | -0.2515 | Yes |
| 201 | 327067\_1 | slc25a4 |  |  | 30176 | -1.326 | -0.2454 | Yes |
| 202 | 100149436\_1 | si:ch211-189a15.5 |  |  | 30256 | -1.369 | -0.2385 | Yes |
| 203 | 100006428 | LOC100006428 |  |  | 30579 | -1.567 | -0.2379 | Yes |
| 204 | 100148818 | si:ch73-55i23.1 |  |  | 30770 | -1.736 | -0.2320 | Yes |
| 205 | 100126128 | zgc:173594 |  |  | 31080 | -2.058 | -0.2276 | Yes |
| 206 | 564295\_2 | traf2a |  |  | 31089 | -2.074 | -0.2137 | Yes |
| 207 | 368481 | stat1b |  |  | 31215 | -2.255 | -0.2022 | Yes |
| 208 | 321622 | hmgb1a |  |  | 31299 | -2.430 | -0.1881 | Yes |
| 209 | 558816 | LOC558816 |  |  | 31393 | -2.629 | -0.1730 | Yes |
| 210 | 403131 | tlr4ba |  |  | 31734 | -3.963 | -0.1566 | Yes |
| 211 | 559768 | zgc:173593 |  |  | 31762 | -4.238 | -0.1284 | Yes |
| 212 | 336426\_1 | chmp1b |  |  | 31821 | -4.782 | -0.0974 | Yes |
| 213 | 550542\_1 | chmp4c |  |  | 31822 | -4.783 | -0.0646 | Yes |
| 214 | 373107\_1 | birc2 |  |  | 31825 | -4.799 | -0.0318 | Yes |
| 215 | 337413 | stat6 |  |  | 31868 | -4.934 | 0.0007 | Yes |
Table: GSEA details [plain text format]

  

Fig 2: NECROPTOSIS(DRE04217)      
 Blue-Pink O' Gram in the Space of the Analyzed GeneSet

  

Fig 3: NECROPTOSIS(DRE04217): Random ES distribution      
 Gene set null distribution of ES for **NECROPTOSIS(DRE04217)**
